# Impact of adherence to healthy habits on the quality of life of cancer survivors: a study from Uruguay

**DOI:** 10.3332/ecancer.2025.1944

**Published:** 2025-07-11

**Authors:** Natalia Camejo, Cecilia Castillo, Nicolas Ayala, Joaquin Manzanares, Gianina Muñoz, Lujan Cabrera, Dahiana Amarillo, María Guerrina, Guadalupe Herrera, Carolina Dörner, Gabriel Krygier

**Affiliations:** 1 Department of Clinical Oncology, Hospital de Clínicas, School of Medicine, Universidad de la República, Montevideo 11600, Uruguay; 2 Departmental Hospital of Soriano ‘Zoilo A. Chelle’, Soriano 75000, Uruguay; 3 Academic Unit of Preventive and Social Medicine, School of Medicine, Universidad de la República, Montevideo 11600, Uruguay; a https://orcid.org/0000-0002-8684-0291; b https://orcid.org/0000-0002-0417-0512; c https://orcid.org/0009-0002-7363-7945; d https://orcid.org/0009-0009-5426-6772; e https://orcid.org/0009-0004-4408-8304; f https://orcid.org/0000-0002-4551-3980; g https://orcid.org/0000-0002-8615-8639; h https://orcid.org/0000-0002-5983-0688; i https://orcid.org/0000-0003-3758-2545; j https://orcid.org/0000-0002-0518-1854

**Keywords:** cancer survivors, quality of life, exercise, healthy lifestyle, health behaviours, breast neoplasms, cancer survivors

## Abstract

**Introduction:**

Healthy habits such as regular physical activity, a balanced diet and tobacco abstinence are associated with better health-related quality of life (HRQoL) in cancer survivors. However, there is limited evidence on this relationship in Latin American countries, where socioeconomic and cultural factors may influence adherence to healthy behaviours.

**Objectives:**

To evaluate the relationship between adherence to healthy lifestyle recommendations and HRQoL in cancer survivors in Uruguay.

**Materials and methods:**

A cross-sectional study was conducted with 241 early-stage cancer survivors treated at two hospitals in Uruguay. Adherence to healthy habits was assessed using a questionnaire based on the American Cancer Society recommendations. HRQoL was measured using the RAND-36 questionnaire. Categorical variables were analysed using the chi-square test, while continuous variables were analysed using Student’s t-test or the Mann–Whitney test. The correlation between SF-36 dimensions and healthy habits was assessed using Spearman’s coefficient.

**Results:**

The median age was 66.7 years and 55.2% were women. The most common cancers were breast (31.1%), colorectal (28.2%) and prostate (26.6%). A total of 62.7% of participants adhered to three or more healthy habits. Adherent patients showed significantly higher HRQoL scores, particularly in physical function (62.68 versus 45.67, *p* < 0.001), energy/fatigue (64.83 versus 45.89, *p* < 0.001) and emotional well-being (69.43 versus 53.02, *p* < 0.001). Adherence to multiple healthy habits was significantly correlated with improvements in physical and mental domains, with energy/fatigue showing the strongest correlation (rs = 0.66, *p* < 0.001).

**Conclusion:**

Adherence to healthy habits has a cumulative positive impact on the HRQoL of cancer survivors. This study highlights the need to design comprehensive interventions to promote healthy behaviours in this population, contributing to the global evidence on cancer care and underscoring its importance in developing countries.

## Introduction

Cancer represents one of the leading causes of death in our country, being responsible for 25% of annual deaths according to statistics from the Ministry of Health [1]. Between 2014 and 2018, 85,336 new diagnoses were registered, with an annual average of 17,067 cases, including non-melanoma skin cancer. Cancer survivors face not only the sequelae of the disease and its treatments, but also an increased risk of developing secondary tumours, metabolic and cardiovascular diseases, in addition to a significant deterioration in their health-related quality of life (HRQoL) [2–5]. This risk is particularly high in older adults, who are also more vulnerable to functional impairment [6–8].

Although many of these consequences can be attributed to the direct effects of cancer or its therapies, lifestyle habits also play a key role in the health status of survivors [9]. This reinforces the importance of promoting healthy behaviours in this population. A fundamental step in this direction is to assess the degree of compliance with the American Cancer Society (ACS) recommendations on healthy lifestyles [10]. International research has shown that a significant proportion of survivors do not follow these guidelines [11–13]. Furthermore, differences between the habits of survivors and those without a history of cancer are often insignificant [11]. Although a cancer diagnosis could be an ‘opportune moment’ to stimulate lifestyle changes, few people manage to implement these transformations effectively [11, 13–15]. This underscores the need to identify strategies to facilitate adherence to healthy behaviours.

Another relevant aspect is the potential interrelationship between different lifestyle behaviours. For example, smoking cessation could motivate a more balanced diet and an increase in physical activity (PA) [16]. Initial studies have found significant correlations between smoking, adequate fruit and vegetable consumption and PA [13, 14], although these investigations are usually limited to specific populations with small sample sizes.

Healthy behaviours, such as moderate or intense PA and a diet rich in plant foods and low in fat, have been shown to improve physical performance in breast and prostate cancer survivors over 60 years of age [10, 16]. In addition, there is evidence that PA is associated with improvements in the physical and functional well-being of patients with colorectal and breast cancer, regardless of their age group [17, 18]. It has also been observed that exercise could reduce the risk of recurrence and mortality in these same types of cancer [19–22].

Finally, preliminary research has shown that survivors who comply with several recommendations simultaneously report better HRQoL compared to those who follow only one [13]. This may be explained by the fact that adopting multiple healthy habits reduces the perception of vulnerability to disease and improves the sense of control over their health, which in turn positively influences their HRQoL [10, 16].

In a recent study conducted by our team, we found that only 28.5% of survivors complied with four or five of the ACS recommendations, while 42% had low adherence (less than three recommendations). Although significant differences in adherence were identified according to variables such as sex, age and educational level, the impact of these behaviours on HRQoL was not analysed. Therefore, this work seeks to address that gap, exploring how healthy habits are associated with HRQoL dimensions in this population.

### Overall objective

To assess the relationship between adherence to ACS recommendations and HRQoL in early cancer survivors using the SF-36 questionnaire.

## Materials and methods

A cross-sectional observational study that included 241 patients over 18 years of age diagnosed with early cancer (stages I–III) attended at the Oncology Services of the Hospital de Clínicas and the Departmental Hospital of Soriano between March 1 and June 1, 2024. Patients with breast, prostate, colorectal, anal canal, bladder, kidney, testicular, ovarian, endometrial, skin melanoma or cervical cancer were included. These cancers were chosen because of their relevance in our setting, their impact on the quality of life of survivors and high long-term survival rates. Participants had to have completed active treatment with curative intent (surgery, chemotherapy and/or radiotherapy) at least 12 months prior to inclusion, and be in regular clinical follow-up without evidence of active disease, i.e., in the control stage. Patients who, at the time of the study, were still receiving hormonal therapy as part of adjuvant treatment (e.g., tamoxifen or aromatase inhibitors in breast cancer or LHRH analogues in prostate cancer) were allowed to be included, provided they had no disease progression or limiting adverse effects. All patients gave informed consent to participate in the study.

Population and data collection: Demographic, clinical and health-related data were collected through the Oncologic Electronic Health Record and by means of a questionnaire specifically designed for this study. Variables such as age at diagnosis, date of diagnosis, tumour topography, stage according to Tumour, Node, Metastasis (TNM) classification origin and educational level were recorded.

Evaluation of healthy habits: Five key habits were evaluated, based on ACS recommendations:

1. PA: Participants reported the number of minutes dedicated weekly to PA. It was considered sufficient to comply with at least 150 minutes of moderate or intense PA.
2. Fruit and vegetable consumption: Participants were asked how many times a day they consumed 100% fruit juice, fruit, green leafy vegetables, potatoes (except french fries) and other vegetables. Participants were classified as consuming five or more servings per day or less.
3. Smoking: Smoking was defined as having smoked at least 100 cigarettes in life and continuing to smoke at present.
4. Nutritional status: Body mass index (BMI) was calculated from reported weight and height, with BMI <30 kg/m² being considered adequate.
5. Alcohol consumption: The frequency and quantity of alcohol consumption in the last 30 days were measured, defined as excessive consumption of more than one drink per day for women and two drinks per day for men or recent episodes of high consumption.

Quality of life assessment: HRQoL was measured using the RAND-36 Health Status Inventory. This measure contains four physical domains (physical functioning, role-physical, bodily pain and general health) and four mental domains (vitality, social functioning, role-emotional and mental health) that can be used to formulate an overall composite health score (i.e., HRQoL), ranging from 0 (worst HRQoL score) to 100 (best HRQoL score) [23]. The RAND-36, a well-validated measure of HRQoL, has been frequently used in cancer survivors [24–28].

### Statistical analysis

Quantitative variables are expressed by their frequencies (absolute and relative percentages). Quantitative variables are expressed by their summary measures. The association between the sociodemographic and disease variables and the dimensions of the SF-36 scale with respect to adherence to healthy habits was assessed by the chi-square test in the case of qualitative variables and by the Student *t* test or Mann–Whitney test in the case of quantitative variables.

The scores per dimension are represented by box-and-line plots.

The correlation between the score of each of the dimensions evaluated in the SF-36 questionnaire and the number of healthy behaviours adopted was analysed using Spearman’s correlation coefficient.

*p* values less than 0.05 indicated statistical significance.

The data were analysed using R software (version 4.4.1).

### Ethical aspects

This study was conducted following international ethical guidelines for biomedical research, in compliance with the ‘MERCOSUR Norms on Regulation of Clinical Studies’ and the ‘Declaration of Helsinki’. In addition, the research regulations approved by the National Ethics Commission in 2019 were respected. Fundamental ethical principles, such as informed consent of patients and confidential treatment of data, were guaranteed to ensure the integrity and well-being of all study participants. In accordance with the Data Protection Law, Law 18.331, measures were taken to preserve the privacy and confidentiality of the information collected, ensuring that personal data are not used during the analysis or disclosed in the dissemination of the results.

## Results

The study included 241 patients diagnosed with stages I–III cancer. The median age was 66.7 years and 55.2% were women. Most of the participants were from the interior of the country (90.9%) and the predominant educational level was high school (49.8%). The most frequent types of cancer were breast (31.1%), colorectal (28.2%) and prostate (26.6%) Table 1. The median time since diagnosis is 41 months (quartile 1 = 23 months; quartile 3 = 71 months, minimum 8 months and maximum 296 months).

Only 16% of the patients consumed enough fruits and vegetables, while 64.7% maintained a BMI of less than 30. Of the patients, 33.6% achieved the recommended PA, 86.7% did not use tobacco and 96.3% did not drink alcohol to excess (Table 2).

37.3% of the patients showed low adherence (less than three guidelines) Table 3.

In terms of sex, no significant differences were found between men and women in terms of adherence (*p* = 0.396). The median age was slightly higher in patients with low adherence (68.14 years) compared to those with high adherence (65.89 years), although this difference did not reach statistical significance (*p* = 0.195). Educational level showed a significant association with adherence (*p* < 0.001), as 67.5% of patients with high adherence had complete secondary or tertiary education, while 64.4% of patients with low adherence had only incomplete secondary education. No significant differences were observed in origin (*p* = 0.428) or median age at diagnosis (*p* = 0.055).

The SF-36 questionnaire demonstrated excellent internal consistency in this population, with a Cronbach’s alpha of 0.957, confirming its reliability for assessing HRQoL in the patients studied.

**Figure 1.**
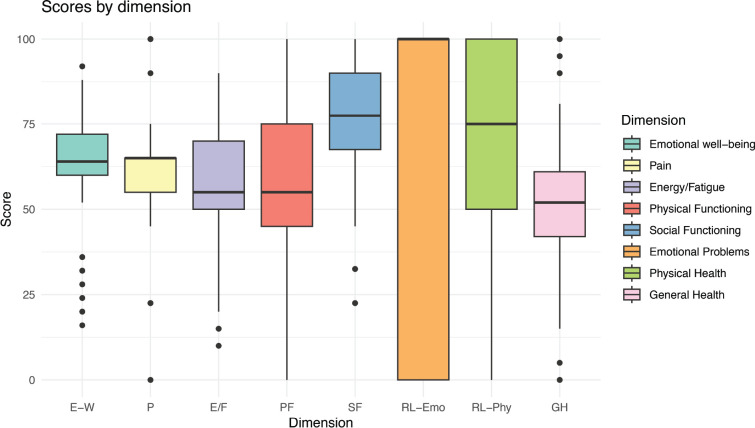
Distribution of SF-36 questionnaire scores (*n* = 241). FF, Physical function; LR-Phys, Limitations in role due to physical health; LR-Emo, Role limitations due to emotional problems; E/F, Energy/Fatigue; BE, Emotional well-being; FS, Social functioning; D, Pain; SG, General health.

The median scores of the RAND-36 questionnaire for the different dimensions assessed are summarised in Figure 1 and Table 4. The medians, interquartile ranges and dispersion of the scores reveal distinctive patterns between the physical and mental domains.

In the physical domains, such as physical function, bodily pain and general health, the medians were lower (approximately 50–60 points), indicating significant limitations in these areas. In contrast, in the mental domains, such as emotional well-being, social function and role limitations due to emotional problems, higher medians were recorded (70–80 points), although the energy/fatigue dimension had a lower median (~60 points) indicating a moderate perception of vitality.

### Relationship between adherence to healthy habits and HRQoL

The scores obtained in the SF-36 questionnaire showed that patients with greater adherence to healthy habits reported better results in the key dimensions of quality of life (Table 4).

The scores obtained in the SF-36 questionnaire showed that patients with greater adherence to healthy habits presented significantly higher values in the physical and mental dimensions (Table 4). In the physical domains, the differences were statistically significant in all the dimensions evaluated, while in the mental domains, the energy/fatigue and emotional well-being dimensions also showed significant differences between adherents and non-adherents.

### Correlation between healthy habits and HRQoL

The Spearman correlation analysis showed significant associations between the number of healthy habits and various SF-36 dimensions, particularly physical functioning, energy, emotional well-being, and pain (Table 5).

## Discussion

Cancer survivors face a significant burden of physical and emotional sequelae resulting from the disease and its treatments. These limitations not only affect their overall health, but also their HRQoL, a critical aspect of ensuring a comprehensive recovery. International research has highlighted that healthy behaviours, such as regular PA and a balanced diet, have a significant impact on the HRQoL of survivors, mitigating the adverse effects of treatments and reducing the risk of recurrence [29, 30]. This study contributes to that evidence, being one of the first investigations in Uruguay to evaluate adherence to healthy habits and their relationship with HRQoL in this population.

Our findings show that scores in the physical domains (physical function, bodily pain and general health) are consistently lower than in the mental domains, reflecting a significant impact of the sequelae of cancer and its treatments. This pattern is consistent with previous studies that have identified physical function and pain as particularly affected dimensions in cancer survivors [6, 16, 29, 30].

Moreover, the significant differences between adherents and non-adherents in these domains underscore the key role of healthy behaviours in physical recovery. Regular PA appears to be the main driver of these improvements, contributing to improved functional capacity and reduced fatigue [6, 30].

In contrast, the mental domains (emotional well-being, social function and emotional role) presented higher scores in general, although significant differences were also observed between adherents and non-adherents in emotional well-being and energy/fatigue. This could reflect not only better emotional management in adherents, but also the positive impact of healthy habits on perceived control over health and emotional resilience. However, the energy/fatigue dimension, although belonging to the mental domains, showed lower scores compared to other domains, highlighting the persistence of this symptom as a key challenge for this population.

This study confirms that adherence to multiple healthy habits has a cumulative effect on improving HRQOL, as has been reported in previous research [4, 6, 29]. Furthermore, our findings reinforce the idea that adopting multiple healthy habits not only improves physical dimensions, but also mental dimensions by reducing the perception of vulnerability and fostering a greater overall sense of well-being [6]. Although cancer survivors attribute the adoption of healthy lifestyles to a reduction in the likelihood of cancer recurrence, [22] it is likely that there are other mechanisms that explain these cumulative benefits. For example, PA has been shown to improve HRQoL by strengthening cardiovascular capacity, muscle strength and perceived physical functioning, while reducing fatigue [4]. In addition, a diet rich in fruits and vegetables, together with abstinence from smoking, is associated with a lower risk of depression, which negatively affects HRQoL [6, 20, 21].

These results suggest that several mechanisms interact to enhance the benefits of these behaviours, improving both the recurrence/mortality outlook and overall quality of life in cancer survivors.

These results highlight the need to implement specific interventions aimed at improving physical function, reducing pain and mitigating fatigue, three of the most affected areas in this population. In addition, promoting strategies that encourage healthy behaviours, such as regular PA and a diet rich in fruits and vegetables, may generate significant benefits in global HRQoL, as suggested by the studies of Blanchard *et al* and Demark-Wahnefried *et al* [4, 16, 17, 29].

It is important to consider that the results obtained correspond to a population of patients under follow-up, at least 12 months after the end of active treatment, and without evidence of active disease or acute toxicity. Therefore, the findings could differ in recently treated patients, in whom the immediate adverse effects of treatment could have a greater impact on quality of life and modulate the effect of healthy habits. Studies such as RENEW [30] and the one published by Mosher *et al* [6] have shown that even several years after treatment, lifestyle interventions continue to have a positive impact, especially in the physical domains of quality of life. However, at earlier post-treatment stages, specific approaches or adaptations of the proposed interventions may be required.

This study provides valuable data as it is one of the first in Uruguay to evaluate adherence to multiple healthy habits and their relationship with HRQoL in cancer survivors. The inclusion of patients from two hospitals improves representativeness, and the use of the RAND-36 questionnaire ensures international comparability of the results. In addition, the pooled analysis of healthy behaviours highlights the importance of multibehavioural strategies to optimise HRQoL, offering clear practical implications for interventions in this population.

However, it has some limitations. The assessment of lifestyle habits was based on self-administered questionnaires, which may introduce recall bias and social desirability. Habits prior to cancer diagnosis, which could influence subsequent changes and their impact on quality of life, were also not analysed. In addition, comorbidities were not considered, which can significantly affect the dimensions assessed and act as confounding factors. These limitations highlight the need for future studies that include objective measures, prospective data and a detailed analysis of comorbidities to better understand the relationship between healthy habits and quality of life in this population.

## Conclusion

This study shows that adherence to healthy habits, especially PA, is significantly associated with improved HRQOL in stages I–III cancer survivors. Adherence to a greater number of lifestyle recommendations cumulatively improves various dimensions of HRQoL, such as physical function, pain, energy/fatigue and emotional well-being. The results underscore the importance of implementing multibehavioural interventions targeting this population, with a comprehensive approach that considers sociodemographic and clinical characteristics to maximise the benefits in quality of life and global health. This study provides relevant evidence to design policies and strategies for health promotion in cancer survivors.

## Conflicts of interest

The authors have no conflicts of interest.

## Funding

The study was not financed.

## Figures and Tables

**Table 1.** Clinicopathological and biological characteristics of the patients (*n* = 241).

Variable		Mean (deviation)
Age		66.7 (13.03)
		***N* (%)**
Sex	Female	133 (55.2)
	Male	108 (44.8)
Educational level	Completed elementary school	23 (9.5)
	Incomplete high school	86 (35.7)
	High school completed	120 (49.8)
	Tertiary	12 (5.0)
Source	Interior	219 (90.9)
	Montevideo	22 (9.1)
Tumor topography	Mama	75 (31.1)
	Colon-rectum	70 (30)
	Prostate	64 (26.6)
	Endometrium	3 (1.2)
	Kidney	9 (3.7)
	Ovary	6 (2.5)
	Testicle	5 (2.1)
	Bladder	4 (1.7)
	Melanoma	3 (1.2)
	Anal canal	1 (0.4)
	Uterus	1 (0.4)
Stadium	I	27 (11.2)
	II	153 (63.5)
	III	61 (25.3)

**Table 2.** Healthy lifestyle behaviours (*n* = 241).

Behavior		Complies with recommendations	
		Yes, *n* (%)	No, *n* (%)
Tobacco use		32 (13.3)	209 (86.7)
Alcohol consumption	Never/Social	88 (36.5)	
	Social	144 (59.8)	
	Excessive		9 ( 3.7)
Consumption of fruits and vegetables	Yes	40 (16.6)	
	No		201 (83.4)
BMI	Normal	89 (36.9)	
	Overweight	67 (27.8)	
	Obesity I	58 (24.1)	
	Obesity II		14 (5.8)
	Obesity III		13 (5.4)
PA	Yes	81 (33.6)	
	No		160 (66.4)

**Table 3.** Number of recommendations adhered to by respondents (*n* = 241).

Number of recommendations adhered to		*n* (%)
	0	2 ( 0.8)
	1	16 ( 6.6)
	2	72 (29.9)
	3	75 (31.1)
	4	53 (22.0)
	5	23 (9.5)

**Table 4.** SF-36 questionnaire scores as a function of adherence to healthy behaviours (*n* = 241).

Dimensions SF 36 scale		Adherent to three or more healthy behaviors		
	Total (*n* = 241)	No (*n* = 90)	Yes (*n* = 151)	*p*
Physical function (mean (deviation))	56.33 (24.55)	45.67 (22.63)	62.68 (23.49)	**<0.001**
Role limitations due to physical health physical health (mean (deviation))	65.98 (32.42)	53.06 (38.19)	73.68 (25.62)	**<0.001**
Role limitations due to emotional problems (mean (deviation))	73.58 (43.76)	75.93 (42.41)	72.19 (44.63)	0.522
Energy/fatigue (mean (deviation))	57.76 (18.45)	45.89 (19.99)	64.83 (13.15)	**<0.001**
Emotional well-being (mean (deviation))	63.30 (15.83)	53.02 (18.01)	69.43 (10.35)	**<0.001**
Social performance (mean (deviation))	71.44 (23.20)	68.08 (26.10)	73.44 (21.12)	0.083
Pain (mean (deviation))	62.50 (21.07)	51.03 (20.13)	69.34 (18.54)	**<0.001**
General health (mean (deviation))	51.85 (21.37)	47.76 (23.46)	54.29 (19.70)	**0.021**

Bold values indicate statistical significance (*p* < 0.05).

**Table 5.** Spearman correlation coefficients, relationship between the score in each dimension of the SF-36 questionnaire and the number of healthy habits (*n* = 241).

Dimensions of the SF-36 questionnaire	r_s_	*p*
Physical function	0.51	**<0.001**
Limitations in the role due to physical health	0.50	**<0.001**
Role limitations due to emotional problems	0.03	0.672
Energy/fatigue	0.66	**<0.001**
Emotional well-being	0.63	**<0.001**
Social functioning	0.17	**0.008**
Pain	0.48	**<0.001**
General health	0.12	0.062

Bold values indicate statistical significance (*p* < 0.05).
